# A Fatal Case of Adenovirus Encephalitis in an Adult With End-Stage Renal Disease

**DOI:** 10.7759/cureus.97156

**Published:** 2025-11-18

**Authors:** Xiong Khee Cheong, Juen Kiem Tan, Ummu Afeera Zainulabid, Najma Kori, Petrick Ramesh Periyasamy

**Affiliations:** 1 Internal Medicine, Universiti Kebangsaan Malaysia Medical Centre, Kuala Lumpur, MYS; 2 Internal Medicine, International Islamic University Malaysia, Kuantan, MYS

**Keywords:** adenovirus infection, antiviral therapy, end-stage renal disease (esrd), new-onset seizure, viral encephalitis syndrome

## Abstract

The spectrum of adenovirus infection can vary from self-limiting mild respiratory illness or gastroenteritis to fatal disseminated disease in immunocompetent hosts. Severe disseminated disease is commonly seen in immunocompromised hosts, including severe pneumonia with acute respiratory distress syndrome, hepatitis, encephalitis, hemorrhagic cystitis, and shock. Meningoencephalitis is a rare manifestation in immunocompetent adults. Early detection of adenovirus infection is necessary as early initiation of appropriate antiviral therapy may prevent life-threatening complications. In this report, we describe a fatal case of adenovirus encephalitis in an adult with end-stage renal disease (ESRD) who presented with a first-onset seizure. The seizure was preceded by a prodromal respiratory illness and septicemic shock, and the diagnosis was confirmed by cerebrospinal fluid (CSF) polymerase chain reaction (PCR) positivity for adenovirus and characteristic electroencephalography (EEG) findings.

## Introduction

Adenovirus is a common cause of self-limiting respiratory, gastrointestinal, and conjunctival infections in immunocompetent hosts. However, in immunocompromised hosts, particularly bone marrow or solid organ transplant recipients and those with advanced HIV, it can lead to fulminant disease. This severe form of adenovirus infection usually presents as severe pneumonia with respiratory failure, encephalitis, or septic shock, which carries a high mortality rate. Adenovirus serotypes 2, 3, 5, 6, 7, and 12 are the typical causative pathogens of severe meningoencephalitis [1]. 

We report a case of life-threatening adenovirus encephalitis in a patient with end-stage renal disease (ESRD) who presented with a seizure. The diagnosis was confirmed by detecting adenovirus DNA in the cerebrospinal fluid (CSF) and identifying typical electroencephalography (EEG) findings. This case highlights the importance of considering adenovirus as a cause of encephalitis in immunocompromised patients and underscores the challenges of using cidofovir in those who are dialysis-dependent.

## Case presentation

A 66-year-old man with a history of hypertension and ESRD on renal replacement therapy via hemodialysis was initially admitted to a district hospital prior to transfer to our care. He was found unconscious in his bathroom at home and subsequently had a tonic-clonic seizure before arriving at the hospital. The patient had a fever and a productive cough for three days prior to admission. On arrival at the emergency department, he was intubated due to a low Glasgow Coma Scale (GCS) score of E1V2M3 [2]. Neurological examination revealed normal tone with downgoing plantar reflexes. Examinations of other systems were unremarkable.

**Figure 1:**
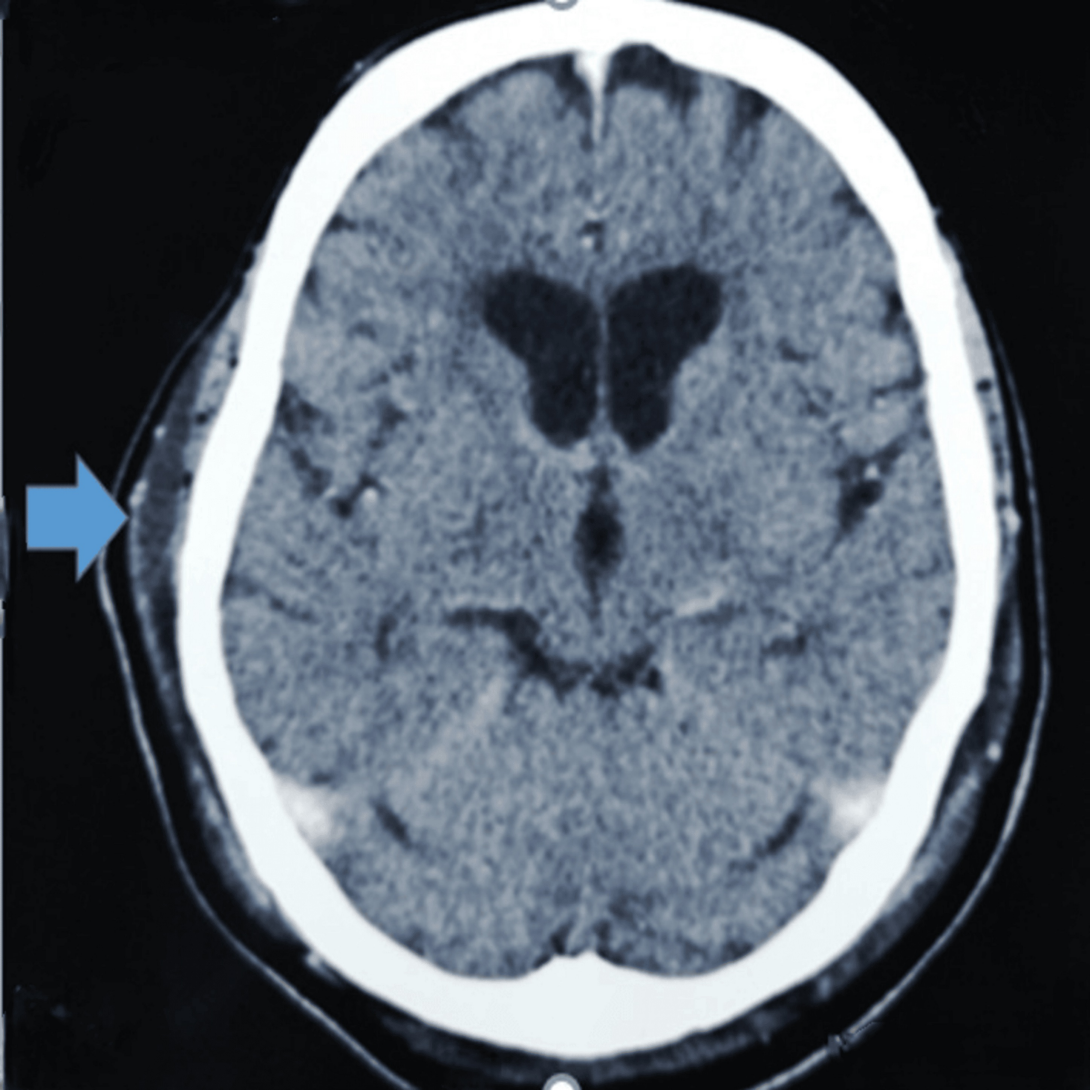
Contrast-enhanced computed tomography scan of the brain showing multifocal old infarcts with right parieto-temporal scalp hematoma (blue arrow).

**Figure 2:**
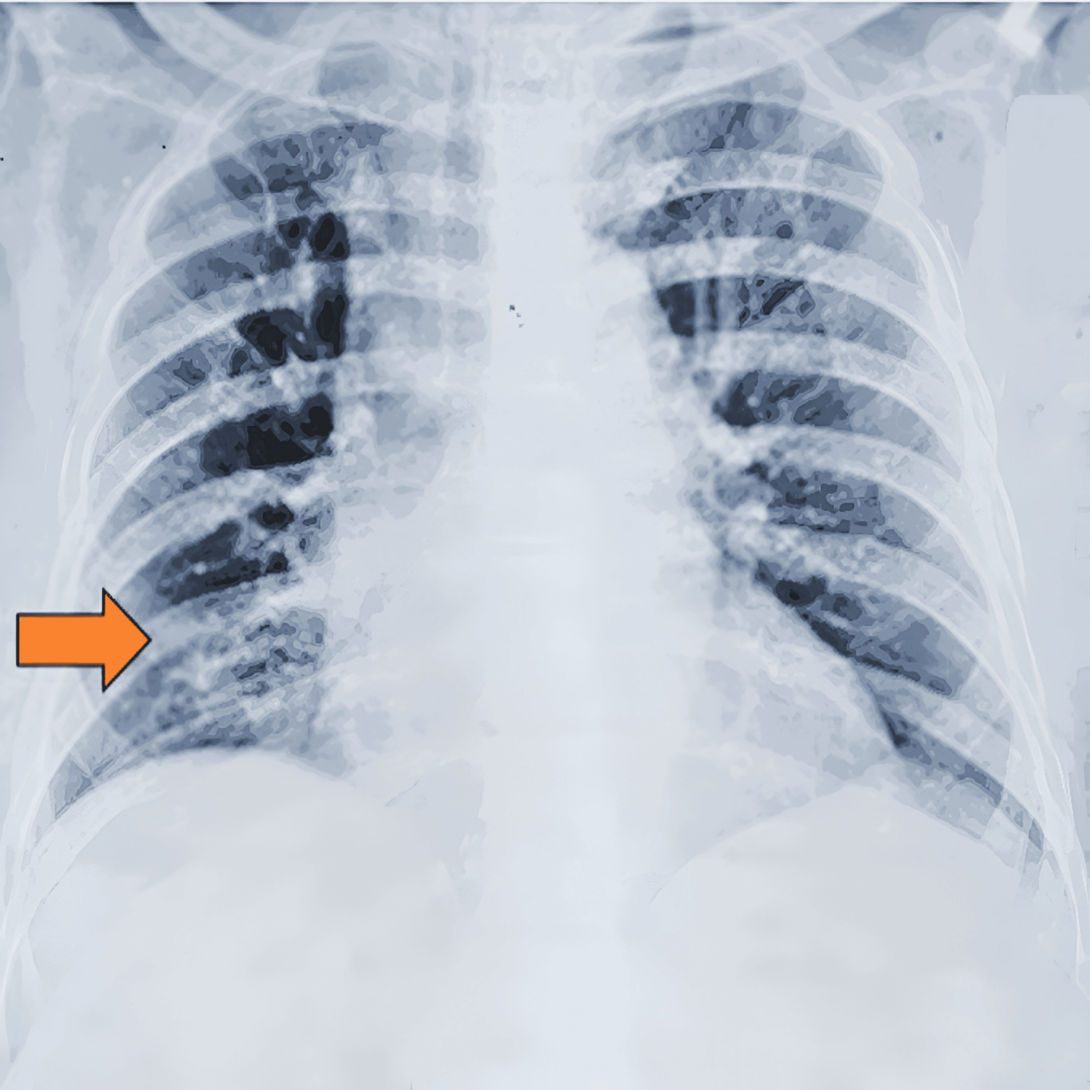
Chest X-ray showing right lower zone consolidation (orange arrow).

The patient was admitted to the intensive care unit (ICU), where he required a ventilator and inotropic support. He had persistent temperature spikes and occasional myoclonic jerks. An initial contrast-enhanced computed tomography (CT) scan of the brain (Figure 1) revealed multifocal old infarcts with a small right parieto-temporal scalp hematoma (post-traumatic, following a fall). A chest X-ray (Figure 2) revealed right lower zone consolidation indicative of pneumonia. A full blood count revealed leukocytosis with a left shift and white cell count of 17.4 x 10^9^/L, predominantly due to neutrophilia. C-reactive protein (CRP) level was also elevated at 1.4 mg/dL. Other laboratory investigations demonstrated normal liver function tests, electrolytes, and coagulation profile.

A lumbar puncture revealed a high opening pressure of 25 cmH_2_O. CSF analysis showed elevated protein (871 mg/L), normal glucose ratio, and elevated white cell count of 10 cells/mm^3^, predominantly lymphocytes. CSF cryptococcal antigen and India ink staining were negative, and culture showed no growth. No other bacterial or fungal pathogens were identified from CSF specimens, and tuberculous polymerase chain reaction (PCR) was negative (Table 1).

**Table 1:** Blood and CSF investigations during hospital admission. PCR: polymerase chain reaction; WBC: white blood cell; ALT: alanine transaminase; ALP: alkaline phosphatase; CSF: cerebrospinal fluid

Parameter	Value	Reference range
Blood		
Hemoglobin (g/dL)	11.4	11.0-16.0
Total WBC count (x 10^9^/L)	17.4	4-10
C-reactive protein (mg/dL)	1.4	<0.5
Sodium (mmol/L)	142	136-145
Urea (mmol/L)	19	3.2-7.4
Creatinine (µmol/L)	840	64-110
ALT (U/L)	20	0-55
ALP (U/L)	88	40-150
CSF		
Opening pressure (cmH_2_0)	25	10-20
Cell count (cells/mm^3^)	10 (predominant lymphocytes)	0-5
Glucose (mmol/L)	6.1	2.2-4.0
Protein (mg/L)	871	150-400
CSF culture	No growth	-
*Mycobacterium tuberculosis *culture	No growth	-
*Mycobacterium tuberculosis *PCR	Negative	-
Adenovirus PCR	Detected	-
Herpes simplex virus 1 & 2 PCR	Not detected	-
Varicella zoster virus PCR	Not detected	-
*Cytomegalovirus *PCR	Not detected	-
*Escherichia coli *K1 PCR	Not detected	-
*Haemophilus influenzae* PCR	Not detected	-
*Listeria monocytogenes *PCR	Not detected	-
*Nisseria meningitidis* PCR	Not detected	-
*Streptococcus pneumoniae* PCR	Not detected	-
*Cryptococcus neoformans/gattii *PCR	Not detected	-
India ink stain	Negative	-
Cryptococcus* *antigen	Negative	-

He was initially treated for meningoencephalitis and started on intravenous ceftriaxone 2 g twice daily and intravenous aciclovir 250 mg once daily. Intravenous phenytoin 100 mg thrice daily and sodium valproate 400 mg twice daily were initiated for seizure control. The antibiotics were escalated to intravenous meropenem due to persistent temperature spikes and myoclonus seizures. The patient showed no clinical improvement after three days of intravenous meropenem. PCR testing then confirmed adenovirus encephalitis (4.5 log10 DNA copies/mL in the CSF). We therefore discontinued intravenous acyclovir and started cidofovir at a renal-adjusted dose of 0.25 mg/kg/week. It should be noted that cidofovir is not widely available in Malaysia. EEG revealed periodic lateralized epileptiform discharges, consistent with viral meningoencephalitis.

After one week of hospitalization, he developed liver impairment with a cholestatic hepatitis pattern: bilirubin 159 µmol/L, alanine transaminase (ALT) 1520 U/L, and alkaline phosphatase (ALP) 458 U/L. The liver impairment was likely multifactorial, caused by antiepileptic drugs (phenytoin and valproate) and hypotension-induced ischemic hepatitis. Blood, fungal, and tracheal cultures remained sterile. His condition continued to deteriorate despite treatment, and he subsequently succumbed to his illness five days following the initiation of cidofovir.

## Discussion

This case report highlights the important need to consider adenovirus meningoencephalitis and illustrates its potentially fatal course in dialysis-dependent patients. It also discusses therapeutic options, including the use of cidofovir in this population.

Our patient was a non-transplant recipient with ESRD who was on hemodialysis. The prodrome of flu-like symptoms with high-grade fever prior to his seizure provided a clue to a viral etiology. The diagnosis of adenovirus meningoencephalitis was confirmed by the isolation of adenovirus DNA in the CSF via PCR, and the EEG showed periodic lateralized epileptiform discharges. The initial diagnosis was challenging, as the presentation mimicked a severe bacterial infection or sepsis.

Adenovirus is a common cause of self-limiting mild upper respiratory tract illness, gastroenteritis, and conjunctivitis in young children and immunocompetent adults. Meningoencephalitis is a rare but fatal manifestation of severe adenovirus infection, with a mortality rate of up to 39% [1]. Severe disseminated infection is often seen in immunocompromised individuals, especially bone marrow or organ transplant recipients and those with AIDS [3-5]. Adenovirus is a rare pathogen in ESRD patients; it is more common in kidney transplant recipients than in those on dialysis [6]. It commonly manifests as hemorrhagic cystitis or acute nephritis [7].

Viral culture is the gold standard for diagnosing adenovirus infection; however, growth may take up to two weeks [8]. CSF PCR for adenovirus DNA is commonly used as it provides a rapid and accurate diagnosis, with a reported 95% sensitivity and 99% specificity [8]. CT or magnetic resonance imaging (MRI) of the brain may be non-specific [9]. EEG is helpful in diagnosing acute encephalitis. In this case, the CSF showed lymphocytosis and elevated protein with a normal glucose concentration, suggestive of viral meningitis. The diagnosis was supported by the detection of adenovirus DNA via PCR in the CSF and EEG findings of periodic lateralized epileptiform discharges.

Currently, there are limited antiviral agents for treating adenovirus infections, with limited data in non-transplant populations. Several agents with in vitro activity against adenovirus, including ganciclovir, ribavirin, cidofovir, and immunoglobulin, have been used with some success [6,7,10,11]. Cidofovir is a broad-spectrum cytosine nucleotide analogue that is effective in vitro against adenovirus. It has been successful in managing adenovirus infection in a stem cell transplant recipient for whom ribavirin had failed [12]. A retrospective study demonstrated favorable outcomes with cidofovir and immunoglobulin in solid organ transplant recipients [13]. Case reports also describe successful treatment of severe adenovirus infection with cidofovir and intravenous immunoglobulin in dialysis-dependent and kidney transplant patients [6,7]. However, cidofovir is associated with nephrotoxicity. There is limited data on cidofovir dosage recommendations for patients undergoing continuous renal replacement therapy [14,15]. However, one study reported that high-flux hemodialysis can result in the renal elimination of up to 50% of the administered dose [16]. Brincidofovir (a lipid conjugate of cidofovir) has been reported to be effective for severe adenoviral disease in transplant recipients without causing nephrotoxicity [17,18]; however, it is not available in our country.

Given the rarity of severe disease in ESRD patients, there is limited literature to guide treatment. We administered a low dose of intravenous cidofovir (0.25 mg/kg/week) in this case because the patient had advanced renal disease and was on renal replacement therapy, and because cidofovir has been associated with better outcomes in disseminated adenovirus infection in previous case reports, despite the limited data available for patients with renal disease. 

## Conclusions

In summary, adenovirus infection is one of the important differential diagnoses of meningoencephalitis, although it is uncommon in immunocompetent adults. Early diagnosis of adenovirus infection is essential, as timely initiation of antiviral therapy may improve outcomes. More studies are required in the future to evaluate the efficacy of cidofovir or brincidofovir therapy for treating adenovirus infections, particularly in individuals with renal insufficiency.

## References

[1] Pham TT, Burchette JL Jr, Hale LP (2003). Fatal disseminated adenovirus infections in immunocompromised patients. Am J Clin Pathol.

[2] Teasdale G, Jennett B (1974). Assessment of coma and impaired consciousness. A practical scale. Lancet.

[3] Awosika OO, Lyons JL, Ciarlini P (2013). Fatal adenovirus encephalomyeloradiculitis in an umbilical cord stem cell transplant recipient. Neurology.

[4] Frange P, Peffault de Latour R, Arnaud C (2011). Adenoviral infection presenting as an isolated central nervous system disease without detectable viremia in two children after stem cell transplantation. J Clin Microbiol.

[5] Schnurr D, Bollen A, Crawford-Miksza L, Dondero ME, Yagi S (1995). Adenovirus mixture isolated from the brain of an AIDS patient with encephalitis. J Med Virol.

[6] Hatlen T, Mroch H, Tuttle K, Ojogho O, Rooney M, Desmond S, Bani-Hani S (2018). Disseminated adenovirus nephritis after kidney transplantation. Kidney Int Rep.

[7] Hofland CA, Eron LJ, Washecka RM (2004). Hemorrhagic adenovirus cystitis after renal transplantation. Transplant Proc.

[8] Buckwalter SP, Teo R, Espy MJ, Sloan LM, Smith TF, Pritt BS (2012). Real-time qualitative PCR for 57 human adenovirus types from multiple specimen sources. J Clin Microbiol.

[9] Jayaraman K, Rangasami R, Chandrasekharan A (2018). Magnetic resonance imaging findings in viral encephalitis: a pictorial essay. J Neurosci Rural Pract.

[10] Yoon BW, Song YG, Lee SH (2017). Severe community-acquired adenovirus pneumonia treated with oral ribavirin: a case report. BMC Res Notes.

[11] Zhao J, Yap A, Wu E, Low CY, Yap J (2020). Severe community acquired adenovirus pneumonia in an immunocompetent host successfully treated with IV cidofovir. Respir Med Case Rep.

[12] Mann D, Moreb J, Smith S, Gian V (1998). Failure of intravenous ribavirin in the treatment of invasive adenovirus infection following allogeneic bone marrow transplantation: a case report. J Infection.

[13] Permpalung N, Mahoney MV, Alonso CD (2018). Adjunctive use of cidofovir and intravenous immunoglobulin to treat invasive adenoviral disease in solid organ transplant recipients. Pharmacotherapy.

[14] Vossen MG, Gattringer KB, Jäger W, Kraff S, Thalhammer F (2014). Single-dose pharmacokinetics of cidofovir in continuous venovenous hemofiltration. Antimicrob Agents Chemother.

[15] Verma A, Vimalesvaran S, Lampejo T, Deep A, Dhawan A (2022). Use of cidofovir in recent outbreak of adenovirus-associated acute liver failure in children. Lancet Gastroenterol Hepatol.

[16] Brody SR, Humphreys MH, Gambertoglio JG, Schoenfeld P, Cundy KC, Aweeka FT (1999). Pharmacokinetics of cidofovir in renal insufficiency and in continuous ambulatory peritoneal dialysis or high-flux hemodialysis. Clin Pharmacol Ther.

[17] Sudhindra P, Knoll B, Nog R, Singh N, Dhand A (2019). Brincidofovir (CMX001) for the treatment of severe adenoviral pneumonia in kidney transplant recipient. Cureus.

[18] Ramsay ID, Attwood C, Irish D, Griffiths PD, Kyriakou C, Lowe DM (2017). Disseminated adenovirus infection after allogeneic stem cell transplant and the potential role of brincidofovir - case series and 10 year experience of management in an adult transplant cohort. J Clin Virol.

